# Kv3.1 and Kv3.4, Are Involved in Cancer Cell Migration and Invasion

**DOI:** 10.3390/ijms19041061

**Published:** 2018-04-02

**Authors:** Min Seok Song, Su Min Park, Jeong Seok Park, Jin Ho Byun, Hee Jung Jin, Seung Hyun Seo, Pan Dong Ryu, So Yeong Lee

**Affiliations:** Laboratory of Veterinary Pharmacology, College of Veterinary Medicine and Research Institute for Veterinary Science, Seoul National University, Seoul 08826, Korea; gan14@snu.ac.kr (M.S.S.); ssumin94@snu.ac.kr (S.M.P.); pjs931@snu.ac.kr (J.S.P.); lljinholl@snu.ac.kr (J.H.B.); elenajin93@snu.ac.kr (H.J.J.); chemikid@snu.ac.kr (S.H.S.); pdryu@snu.ac.kr (P.D.R.)

**Keywords:** tumor hypoxia-related Kv channels, cell density, cell migration and invasion, BDS, cancer metastasis

## Abstract

Voltage-gated potassium (Kv) channels, including Kv3.1 and Kv3.4, are known as oxygen sensors, and their function in hypoxia has been well investigated. However, the relationship between Kv channels and tumor hypoxia has yet to be investigated. This study demonstrates that Kv3.1 and Kv3.4 are tumor hypoxia-related Kv channels involved in cancer cell migration and invasion. Kv3.1 and Kv3.4 protein expression in A549 and MDA-MB-231 cells increased in a cell density-dependent manner, and the pattern was similar to the expression patterns of hypoxia-inducible factor-1α (HIF-1α) and reactive oxygen species (ROS) according to cell density, whereas Kv3.3 protein expression did not change in A549 cells with an increase in cell density. The Kv3.1 and Kv3.4 blocker blood depressing substance (BDS) did not affect cell proliferation; instead, BDS inhibited cell migration and invasion. We found that BDS inhibited intracellular pH regulation and extracellular signal-regulated kinase (ERK) activation in A549 cells cultured at a high density, potentially resulting in BDS-induced inhibition of cell migration and invasion. Our data suggest that Kv3.1 and Kv3.4 might be new therapeutic targets for cancer metastasis.

## 1. Introduction

Tumor hypoxia is a characteristic of cancer that differs from normal tissue. Because of the rapid growth of tumors, the blood supply cannot provide sufficient oxygen to tumor cells, and as a result, the cells encounter a hypoxic microenvironment. Tumor cells alter their metabolism and increase their migratory and metastatic behavior to overcome this hypoxic microenvironment [1]. Furthermore, hypoxia is associated with extracellular matrix remodeling, which plays crucial roles in metastasis [2]. During these changes, cells are forced to induce genomic and proteomic changes that may contribute to tumor malignancy [3,4,5].

Hypoxia induces depolarization of membrane potential by inhibiting the activity of several oxygen-sensitive K^+^ channels, including voltage-gated potassium (Kv) channels, Ca^2+^-activated K^+^ channels, and the two-pore domain TASK-like K^+^ channels [6,7]. Specifically, the expression and roles of Kv channels, as oxygen-sensitive channels, have been well investigated. Hypoxia inhibits Kv channel activity in various systems, such as carotid body and pulmonary artery myocytes, where the oxygen regulation is important [8,9,10]. During hypoxia, hypoxia-inducible factors (HIFs) appear to be important for regulating O_2_-sensing machinery and HIF-1α regulates K^+^ channel activity [7,11,12,13]. HIF-1α is one of the most important transcription factors for controlling many hypoxia-inducible genes [14], and accumulating evidence suggests that hypoxia-inducible factor-1α (HIF-1α) mediates tumor metabolic responses and promotes tumor proliferation, angiogenesis, and metastasis [15,16,17].

Reactive oxygen species (ROS), ions or molecules that have a single unpaired electron in their outermost shell of electrons are produced as an inevitable byproduct of oxidative phosphorylation in mitochondria. Elevated ROS levels have been detected in almost all cancers; these high levels can result from abnormally increased cellular activities of cancer cells, including metabolic activity and cellular receptor signaling [18,19,20]. ROS promote many aspects of tumor development and progression and also activate certain signaling molecules, including extracellular signal-regulated kinase (ERK) [19,21], which is linked to the regulation of tumor cell proliferation, migration, and invasion [19,21,22,23]. Tumor cells express increased levels of antioxidant proteins to protect themselves against ROS, suggesting that the homeostasis of the intracellular ROS levels is important for cancer cell function [20]. Certain Kv channels, including Kv3.3 and Kv3.4, are known as oxidation-sensitive channels because the oxidation of a cysteine residue at the amino terminus of the channels interrupts their capacity for rapid inactivation due to the formation of a disulphide bond, consequently increasing their electrophysiological function [6,24]. Therefore, these channels function as an oxidation sensor in tumor cells with high ROS levels.

The roles of Kv channels in cancer development and progression have been well investigated. Kv channels are not only involved in cell proliferation and tumor growth but also cell migration, adhesion and metastasis [25,26]. However, mechanistic studies of the functions of Kv channels have not yet been able to clearly explain all the observed phenomena. In fact, both canonical ion permeation-dependent and noncanonical ion permeation-independent processes, including signaling cascades, have been proposed to participate in cancer development and progression [25]. Therefore, new cancer research paradigms that include Kv channels as therapeutic targets may be necessary to more clearly explain the observed phenomena.

## 2. Results

### 2.1. Increases in HIF-1α and ROS Levels Related to Increased Cell Density

**Figure 1:**
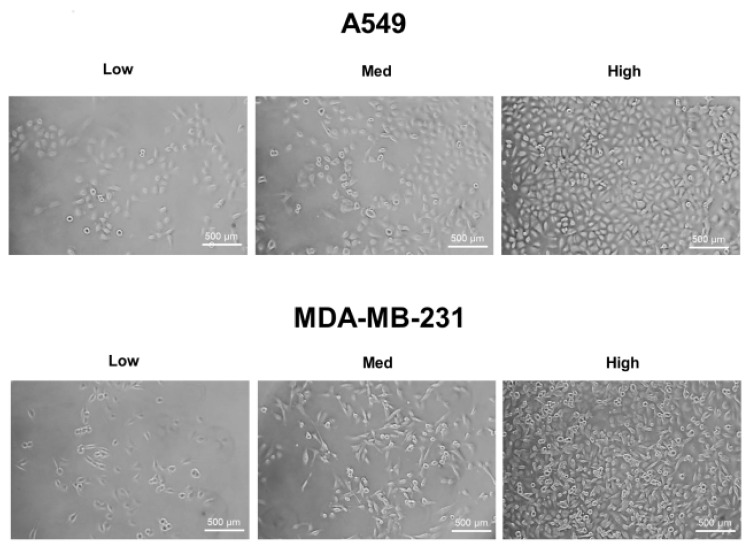

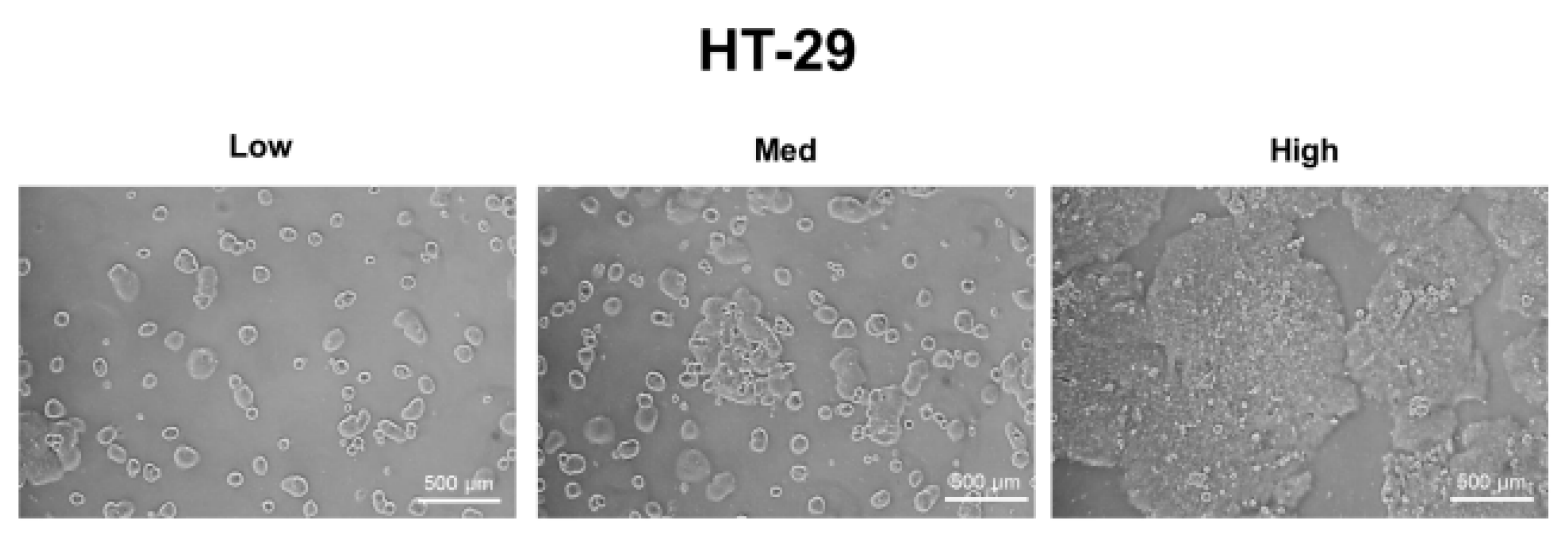
The three cell confluency conditions of A549, MDA-MB-231, and HT-29 cells. A549, MDA-MB-231, and HT-29 cells were grown until they reached the appropriate cell densities. Low cell density was defined as approximately 20~30% cell confluency, medium density as approximately 40~60% cell confluency, and high density as approximately 80~90% cell confluency. Representative images of each cell condition are shown in the figure.

A549, MDA-MB-231, and HT-29 cells were cultured until they reached optimal cell confluency. A low cell density was defined as approximately 20~30% cell confluency, a medium cell density was approximately 40~60% cell confluency, and a high cell density was over 80% confluency. The representative cell-seeding conditions for each of the cell lines are presented in Figure 1.

**Figure 2:**
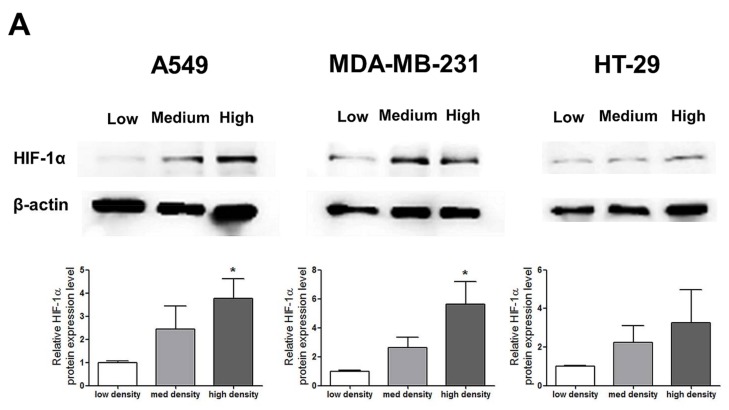

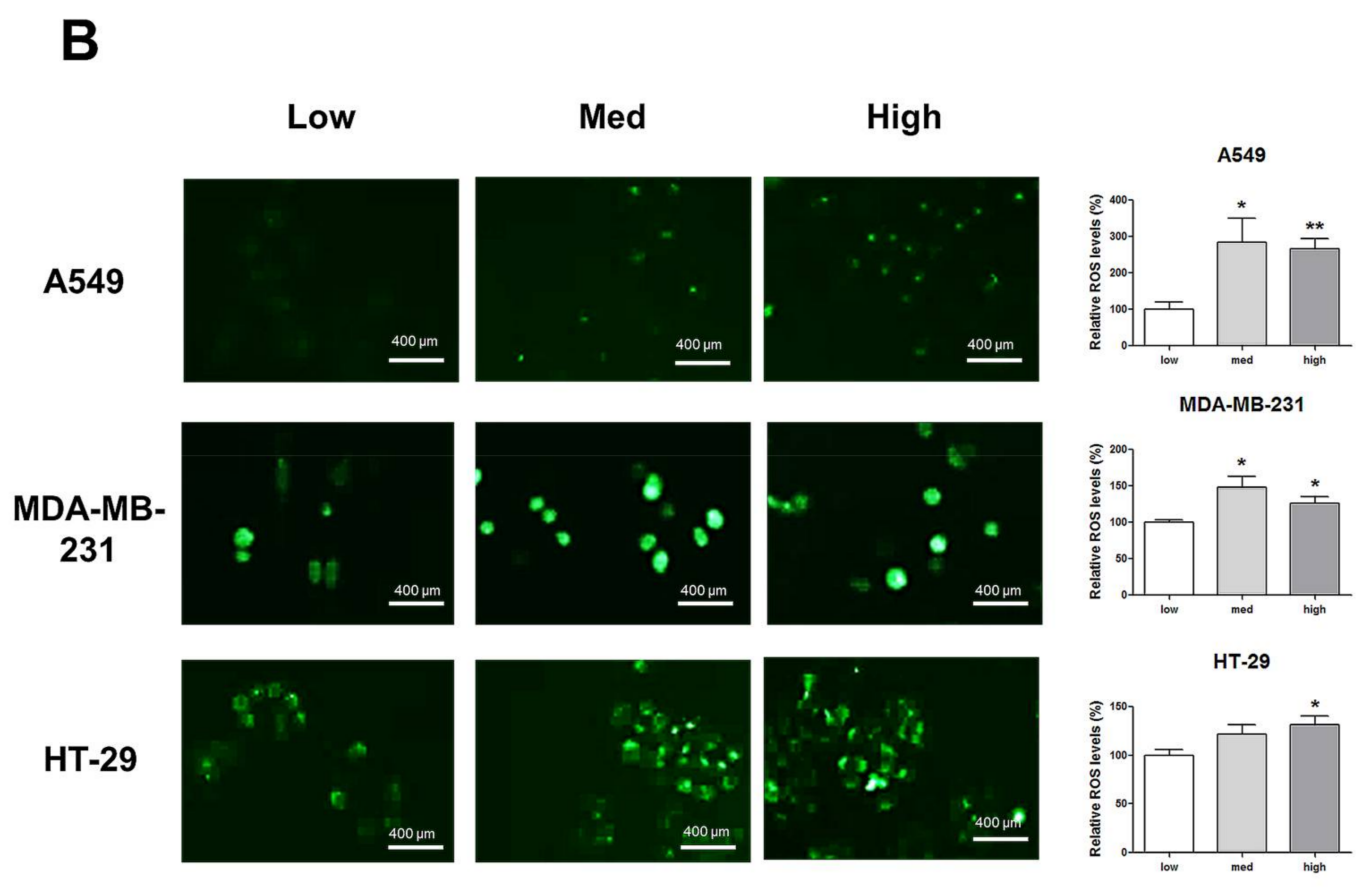
Increased pericellular hypoxia and ROS levels according to the increase in cell density. (**A**) Western blot data demonstrate that HIF-1α expression, which was considered to be induced by pericellular hypoxia in our experiments, was significantly higher in high-density than in low-density in A549 and MDA-MB-231 cells, whereas HIF-1α expression was not increased in high-density compared with that in low-density in HT-29 cells. (**B**) Representative images show increased ROS signals (green) according to the increase in cell density. The graphs show the quantitative analysis of the ROS data. ROS levels were significantly increased with an increase in cell density in A549 and MDA-MB-231 cells, whereas ROS levels in HT-29 cells were significantly increased only when the cells were cultured at a high density. All experiments were performed in triplicate, and the data represent the mean ± standard error. * *p* < 0.05 and ** *p* < 0.01 versus the low-density value.

HIF-1α expression, which represents cell density-related pericellular hypoxia, increased approximately four-fold and six-fold in A549 and MDA-MB-231 cells, respectively, according to the increase in cell density, whereas HIF-1α expression did not significantly increase in HT-29 cells (Figure 2A). ROS levels also increased in A549 and MDA-MB-231 cells according to the increase in cell density. In HT-29 cells, only cells cultured at a high density showed significantly higher ROS levels than the low-density cultured cells (Figure 2B).

### 2.2. mRNA and Protein Expression Changes According to Increased Cell Density

**Figure 3:**
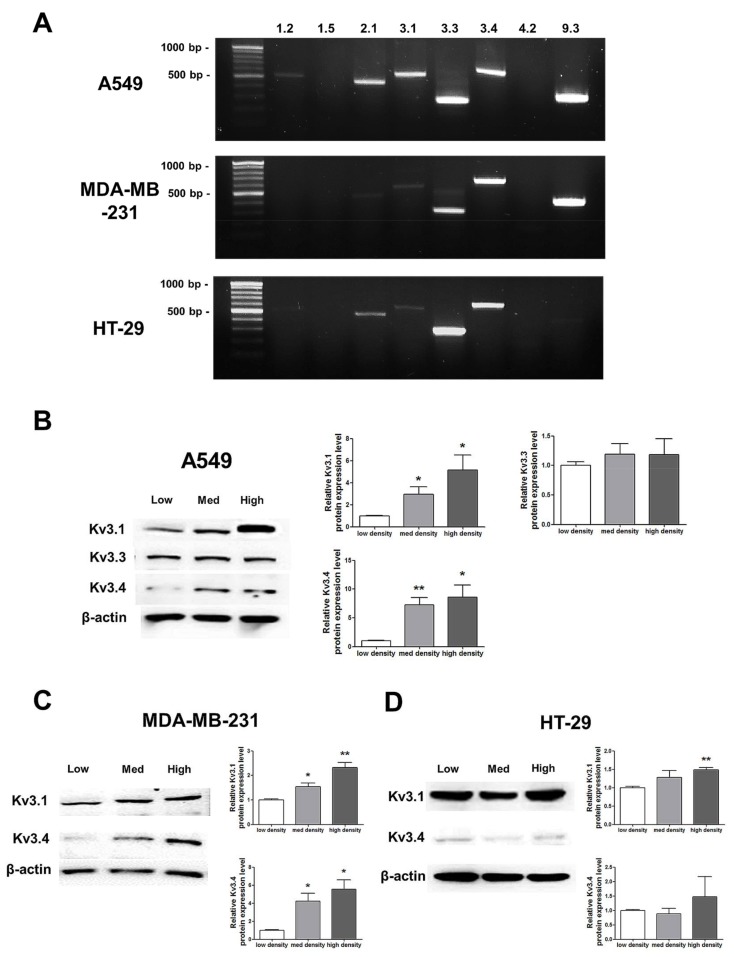
Changes in mRNA and protein expression of Kv3.1, Kv3.3, and Kv3.4 according to cell density. (**A**) RT-PCR data demonstrating that Kv3.1, Kv3.3, and Kv3.4 mRNA was expressed in A549, MDA-MB-231, and HT-29 cells. (**B**) The protein expression levels of Kv3.1, Kv3.3, and Kv3.4 were analyzed by Western blot. Kv3.1 and Kv3.4 were increased in A549 cells dependent on the cell density, whereas Kv3.3 was not altered according to the cell density. (**C**,**D**) Kv3.1 and Kv3.4 protein expression levels were analyzed in MDA-MB-231 and HT-29 cells by Western blot. Kv3.1 and Kv3.4 were increased in MDA-MB-231 cells according to the increase in cell density. Only Kv3.1 was significantly increased in high-density HT-29 cells compared to that in low-density HT-29 cells. Kv3.4 expression was not significantly increased in HT-29 cells as the cell density increased. All experiments were performed in triplicate, and the data represent the mean ± standard error. * *p* < 0.05 and ** *p* < 0.01 versus the low-density value.

RT-PCR analysis demonstrated that among the 8 oxygen-sensitive Kv channels [6], Kv3.1, Kv3.3, and Kv3.4 were highly expressed in A549, MDA-MB-231, and HT-29 cells (Figure 3). Even though several Kv channels, including Kv1.2, Kv2.1, and Kv9.3, were also expressed in the cell lines, the three Kv3 subfamilies were commonly and stably expressed in all of the cell lines (Figure 3A). The Kv3.1 and Kv3.4 protein expression levels were increased in a cell density-dependent manner in A549 cells (Figure 3B). However, Kv3.3 protein expression in A549 cells was not altered by cell density (Figure 3B). Therefore, we decided to focus on the Kv3.1 and Kv3.4 protein expression levels in the other two cell lines. We also observed the same increase in the Kv3.1 and Kv3.4 expression levels according to cell density in MDA-MB-231 cells (Figure 3C). However, in HT-29 cells, Kv3.1 expression was only increased in the high-density cells and not in those cultured at a medium density (Figure 3D). Interestingly, unlike Kv3.1 in A549 and MDA-MB-231 cells, Kv3.4 expression was not increased in HT-29 cells in a cell density-dependent manner (Figure 3D).

### 2.3. The Effect of BDS-II-Mediated Kv3.1 and Kv3.4 Inhibition on Cell Proliferation, Migration, and Invasion

**Figure 4:**
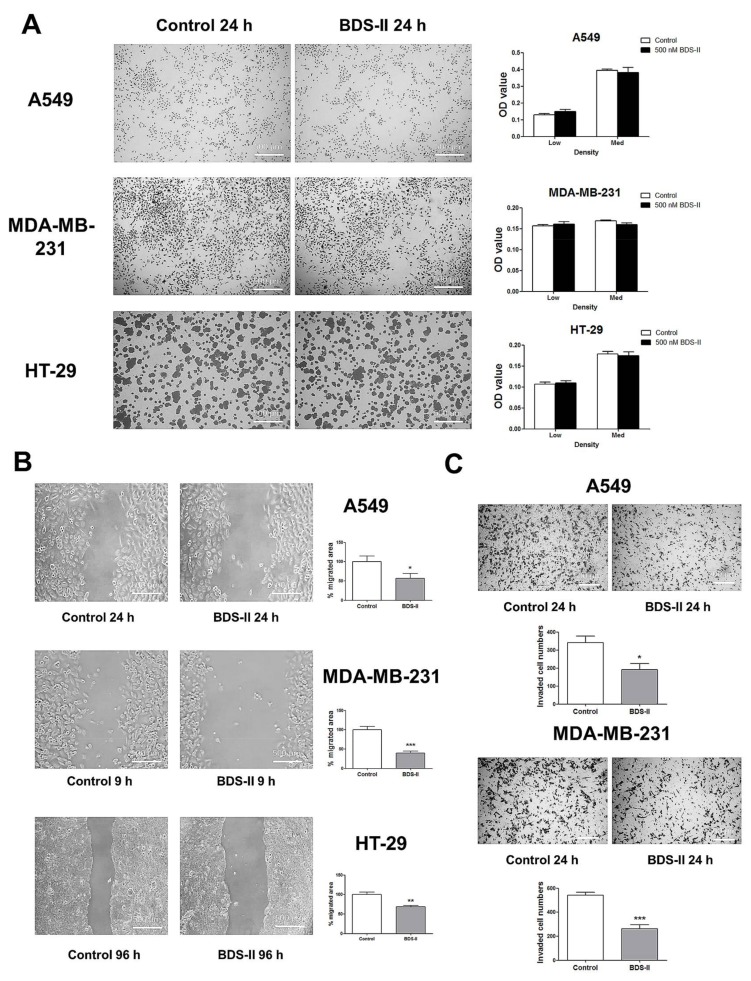
Effect of BDS-II on cell proliferation, migration, and invasion. (**A**) Representative Hemacolor^®^ rapid staining images demonstrate that 24 h of BDS-II (500 nM) treatment did not affect the proliferation of A549, MDA-MB-231, and HT-29 cells. The MTT data also demonstrated that BDS-II did not affect cell proliferation in the three cell lines. (**B**) Representative images demonstrate that 500 nM BDS-II significantly inhibited migration by almost half in A549, MDA-MB-231, and HT-29 cells. (**C**) Hemacolor^®^ rapid staining images demonstrate that the number of cells that migrated through the membrane was reduced in A549 and MDA-MB-231 cells by 500 nM BDS-II treatment. All experiments were performed in triplicate or quadruplicate, and the data represent the mean ± standard error. * *p* < 0.05, ** *p* < 0.01, and *** *p* < 0.001 versus the control value.

We investigated the effect of blood depressing substance (BDS) on cell proliferation and cell movement. Cells cultured at a low or medium density were tested to investigate the effect of 500 nM BDS-II on cell proliferation, and we did not observe an effect of BDS-II on cell proliferation in A549, MDA-MB-231, or HT-29 cells (Figure 4A). However, we found that 500 nM BDS-II affected cell migration and invasion. After 24 h of BDS-II treatment, the cell migration area was reduced by almost half in A549, MDA-MB-231, and HT-29 cells compared with that in the control group (Figure 4B). Cell migration was also inhibited by knockdown of Kv3.4, a specific target of BDS-II, using siRNA in A549 cells, whereas Kv3.1 downregulation did not have any effect on cell migration (supplementary data Figure S1B,F). The number of invasive cells was significantly reduced by 500 nM BDS-II in A549 and MDA-MB-231 cells (Figure 4C). Knockdown of Kv3.1 or Kv3.4 also efficiently inhibited A549 cell invasion (supplementary data Figure S1C,G). However, we observed almost no invasive cells in the HT-29 cultures, even though we used Matrigel in our experiments.

### 2.4. Cell Density-Dependent Kv Channel Expression and the Effect of BDS-II on L-132 Cells

**Figure 5:**
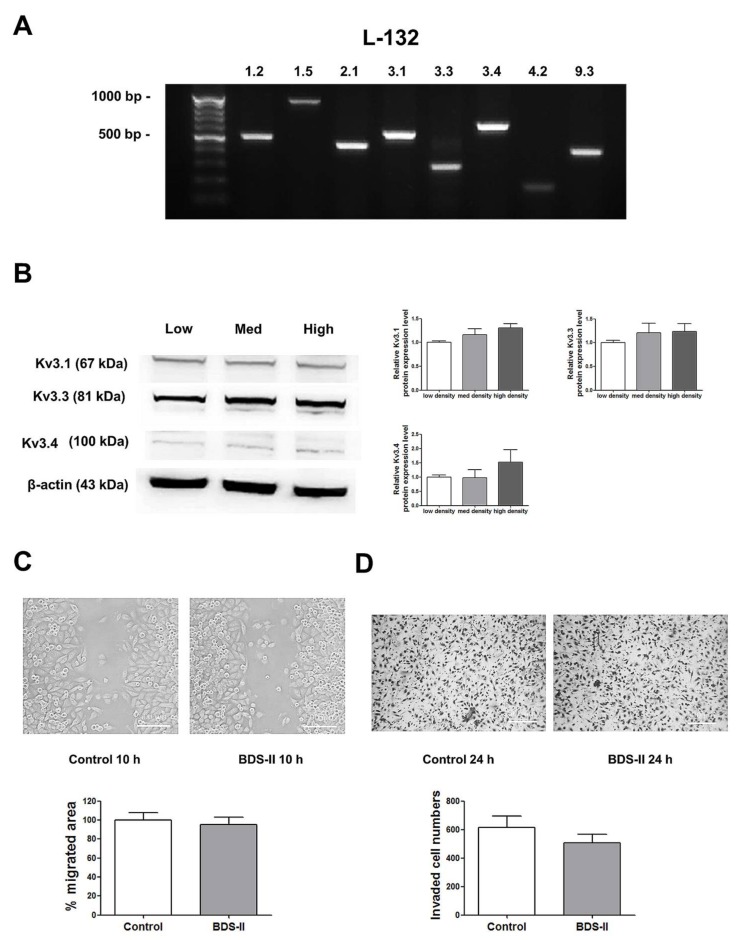
The expression of Kv3.1, Kv3.3, and Kv3.4 and the effect of BDS-II on L-132 cells. (**A**) RT-PCR data demonstrate that L-132 cells express all of the 8 oxygen-sensitive Kv channels at the mRNA level. (**B**) The protein expression levels of Kv3.1, Kv3.3, and Kv3.4 were analyzed by Western blot in L-132 cells. The expression of all three Kv channels was not significantly altered according to the cell density. (**C**) Representative images demonstrate that 500 nM BDS-II did not affect L-132 cell migration. (**D**) Hemacolor^®^ rapid staining images demonstrate that the number of L-132 cells that migrated through the membrane was not altered by 500 nM BDS-II treatment. All experiments were performed in quadruplicate, and the data represent the mean ± standard error.

Next, we determined whether the phenomena we observed were cancer specific. We performed the same experiments using L-132 cells, a normal human lung cell line. RT-PCR analysis demonstrated that L-132 cells also highly express Kv3.1, Kv3.3, and Kv3.4 (Figure 5A); however, unlike A549 cells, the expression levels of Kv3.1, Kv3.3, and Kv3.4 were not increased according to cell density in L-132 cells (Figure 5B). In addition, 500 nM BDS-II did not have an effect on L-132 cell migration and invasion (Figure 5C,D).

### 2.5. Cell Density-Dependent Alterations in the Diameter of Acidic Compartments in A549 and L-132 Cells

**Figure 6:**
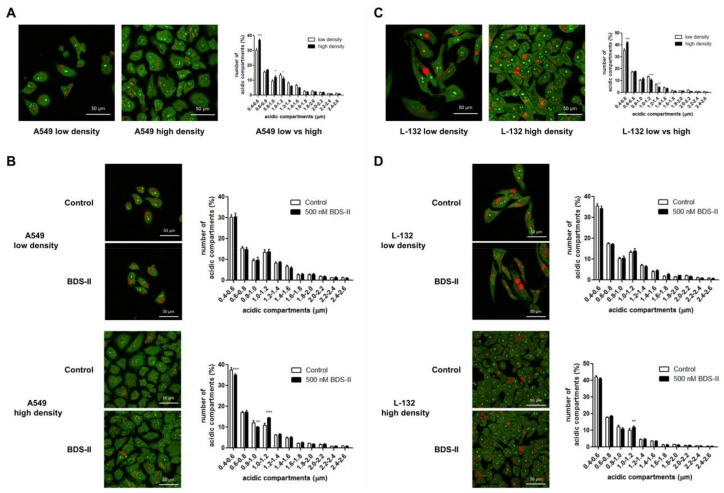
Acridine orange staining represents alterations in the diameter of acidic compartments in A549 and L-132 cells according to cell density. The orange-red fluorescence of acridine orange staining demonstrates the acidic compartments, including the intracellular organelles, and the yellow-green color indicates a slightly acidic or neutral pH. (**A**) The number of small (0.4–0.6 μm) acidic compartments in A549 cells was significantly increased and the number of large (1–1.2 μm) acidic compartments in A549 cells was decreased when the cells reached a high density compared to that of A549 cells at a low density. (**B**) When A549 cells were incubated with 500 nM BDS-II for 30 h, BDS-II had no effect on A549 cells cultured at a low density (*n* = 13), whereas the same concentration of BDS-II decreased the number of small (0.4–0.6 and 0.8–1.0 μm) acidic compartments and increased the number of large (1–1.2 μm) acidic compartments in A549 cells cultured at a high density (*n* = 17). (**C**) The number of small acidic compartments in L-132 cells was also significantly increased when the cells reached a high density compared with that of L-132 cells cultured at a low density. (**D**) L-132 cells were also incubated with 500 nM BDS-II for 30 h, and BDS-II had no effect on the size of the acidic compartments in L-132 cells cultured at either a low (*n* = 13) or high density (*n* = 17). All experiments were repeated the indicated number of times, and the data represent the mean ± standard error. * *p* < 0.05, ** *p* < 0.01, and *** *p* < 0.001 versus the control value.

We found that the number of small (0.4–0.6 μm) acidic compartments [27] in A549 and L-132 cells was significantly increased and that the number of large (1–1.2 μm) acidic compartments in A549 and L-132 cells was decreased when the cells reached high density compared with those of the low-density cells (Figure 6A,C). Therefore, we investigated how BDS-II affects this cell density-dependent alteration. When A549 cells were cultured at a low density, BDS-II did not have an effect on the intracellular acidic compartments in A549 cells, whereas when we cultured A549 cells at a high density, 500 nM BDS-II decreased the number of small (0.4–0.6 and 0.8–1.0 μm) acidic compartments and increased the number of large (1–1.2 μm) acidic compartments (Figure 6B). In contrast, 500 nM BDS-II had little effect on L-132 cells cultured at either a low or high density (Figure 6D).

### 2.6. Effect of BDS-II on ERK Activation According to Increased A549 and L-132 Cell Density

**Figure 7:**
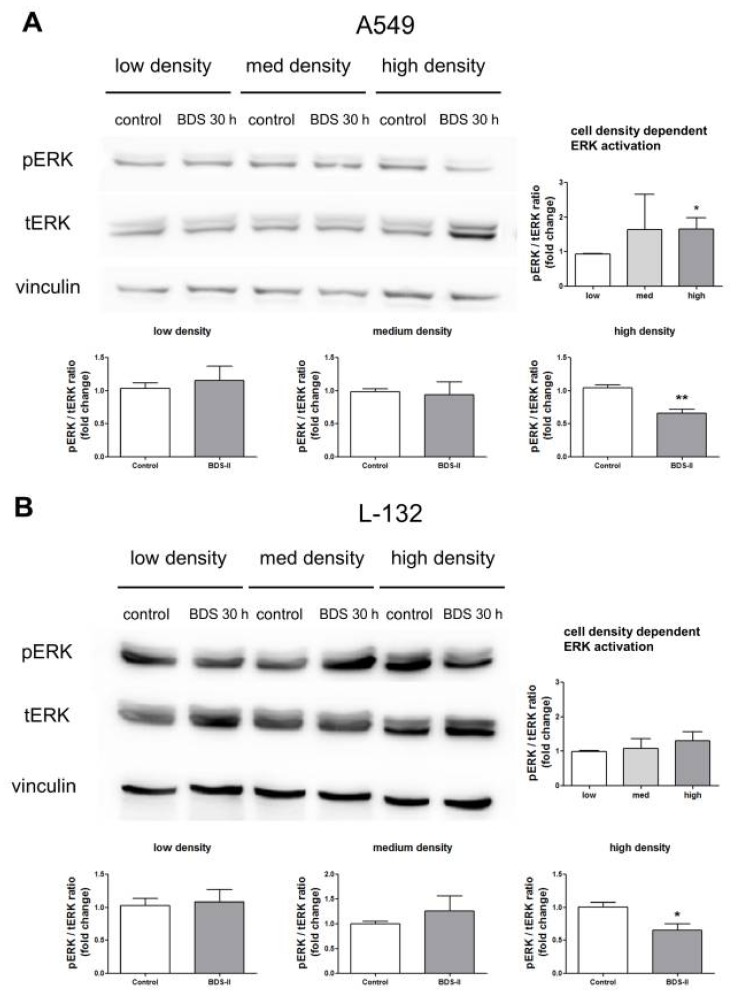
Effect of BDS-II on ERK activation according to the increase in cell density. ERK activation according to the increase in cell density was analyzed in A549 and L-132 cells using the ratio of phosphorylated ERK (pERK) to total ERK (tERK) protein expression. (**A**) Quantitative data demonstrate that ERK was activated in A549 cells cultured at a high density compared with A549 cells cultured at a low density. ERK activation in medium-density A549 cells did not significantly differ from that in low-density A549 cells. (**B**) ERK activation was not altered according to the cell density in L-132 cells. When the cells were incubated with 500 nM BDS-II for 30 h, ERK activation in the high-density A549 or L-132 cells was inhibited. BDS-II had no effect on ERK activation in A549 or L-132 cells cultured at either a low or medium density. All experiments were performed in triplicate, and the data represent the mean ± standard error. * *p* < 0.05 and ** *p* < 0.01 versus the low-density or control value.

We found that ERK activation was higher in high-density cultured A549 cells compared with that in low-density cultured A549 cells (Figure 7A), whereas L-132 cells showed no cell density-related changes in ERK activation (Figure 7B). BDS-II had no effect on ERK activation when either A549 or L-132 cells were cultured at a low or medium density; however, when A549 or L-132 cells were cultured at a high density, BDS-II inhibited ERK activation (Figure 7A,B). Although ERK was activated in a cell density-dependent manner only in A549 cells, BDS-II (500 nM) treatment induced the same effect in both cell lines. Knockdown of Kv3.1 or Kv3.4 using siRNA also inhibited ERK activation (supplementary data Figure S1D,H).

## 3. Discussion

The microenvironment induces broad changes in gene and protein expression, metabolism, and extracellular matrix remodeling, and in turn, tumor cells may be forced to undergo metastasis, which involves escape from the primary tumor, seeding at distinct sites and cell growth [1,2,16,28]. Metastasis is suggested to represent an integrated strategy for cancer cells to avoid oxidative damage and excessive ROS accumulation in the primary tumor site [29].

Our data demonstrated a close relationship between several oxygen-sensitive Kv channel subunits and tumor hypoxia, as well as the importance of these channels in cancer progression. Kv3.1, Kv3.3, and Kv3.4 belong to the Kv3 subfamily, which is a class of Kv channels characterized by positively shifted voltage dependencies and very fast deactivation rates [30], and these Kv channels were examined as tumor hypoxia-related Kv channels in A549, MDA-MB-23, and HT-29 cells, which are highly aggressive lung, breast, and colon cancer cell lines, and all three cell lines expressed Kv3.1, Kv3.3, and Kv3.4. We found a cell density-dependent increase in HIF-1α in A549 and MDA-MB-231 cells, whereas HIF-1α was not significantly increased in a cell density-dependent manner in HT-29 cells, which grow in an aggregate form. Interestingly, Kv3.1 and Kv3.4 protein expression showed almost the same pattern as HIF-1α in all three cell lines, suggesting that Kv3.1 and Kv3.4 are tumor hypoxia-related Kv channels. In addition, we also assumed that the cell density-dependent increases in Kv3.1 and Kv3.4 expression are a tumor cell-specific property because Kv3.1 and Kv3.4 were not increased in a cell density-dependent manner in L-132 cells, a normal cell line.

HIF-1α expression is increased by oxygen depletion or the balance between oxygen and oxidative stress [31,32,33]. Based on our results, we hypothesize that the same expression pattern between HIF-1α and the two Kv channels was caused by cell density-induced pericellular hypoxia. Furthermore, considering that HIF-1α has been reported to regulate Kv currents during chronic hypoxia or the mitochondrial pathway normally used for oxygen sensing [11,34], we concluded that accumulated HIF-1α during cell growth might induce the increase in Kv3.1 and Kv3.4. In HT-29 cells, HIF-1α levels were not significantly increased with an increase in cell density. We hypothesized that the HIF-1α accumulation induced by pericellular hypoxia might already be present in HT-29 cells because of their aggregated form [35]. The comparatively slight increase in the ROS level in HT-29 cells with the increase in cell density can also be interpreted in this scheme. Interestingly, despite the pre-existence of pericellular hypoxia, we observed a cell density-dependent increase in Kv3.1 protein expression when we seeded HT-29 cells at a high density.

Next, we used BDS-II, a Kv3 subfamily-specific blocker, to confirm whether the increased expression of Kv channels had a potential role in cell density regulation by avoiding hypoxia or oxidative damage during cell growth. When we treated cells with BDS-II, cancer cell migration and invasion were inhibited in all three cell lines without an effect on cancer cell proliferation. Cell migration and invasion are important in the initial steps of cancer metastasis, and metastatic cancer cells must go through several steps to spread to new parts of the body; cancer cells must break away from the primary tumor and enter the bloodstream by invading the blood vessels. Therefore, according to the data, Kv3.1 and Kv3.4, which are specific targets of BDS-II, may be good biomarkers and therapeutic targets for cancer metastasis. In fact, Kv3.4 has already been suggested to be a strong biomarker candidate for predicting the malignant progression of laryngeal epithelial precursor lesions [36] or head and neck squamous cell carcinomas [37].

Cells cultured at a high-density exhaust medium faster than cells cultured at a low density, which may cause a rapid drop in pH due to lactic acid production, a by-product of cellular metabolism; therefore, cells cultured at a high density are forced to adapt to microenvironmental alterations in pH. Our data demonstrate that cells cultured at a high density have a higher proportion of small acidic compartments than cells cultured at a low density. BDS-II increased the size of the acidic compartments in high-density cultured A549 cells, and the altered size of the acidic compartments seemed to be similar to the acidic compartments in A549 cells cultured at a low density. However, BDS-II had no effect on the size of the acidic compartments in A549 cells cultured at a low density or in L-132 cells. Considering that regulation of acidic compartments, including lysosomes and acidic vacuoles, is important for cancer progression and adaptation to alterations in the extracellular environment [38,39], our data suggest that BDS-II only inhibited the cell density-dependent microenvironmental adaptation of A549 cells. Furthermore, ROS levels and the two tumor hypoxia-related Kv channels were similarly increased according to cell density in all three cancer cell lines. As mentioned above, cancer may metastasize to avoid oxidative damage and excessive ROS accumulation at the primary tumor site [29]. Along this line of speculation, we suggest that Kv3.1 and Kv3.4 may be sensors of a poor microenvironment around cancer cells and that an increase in the expression of these channels forces cancer cells to initiate migration or invasion.

ROS levels were increased in a cell density-dependent manner in the three cancer cell lines we investigated and ROS induce ERK activation [19,21]. Therefore, we could infer that ERK activation, induced by ROS production during cell growth, initiates cell migration or invasion to avoid oxidative damage and excessive ROS accumulation. Based on this scheme, we hypothesized that BDS might inhibit cell migration and invasion by interrupting ERK activation along with the increase in cell density. ERK activation in high-density cultured normal cells differs from that in malignant tumour cells. ERK activation is inhibited by cell-cell contact inhibition in non-tumorigenic cells [40,41], but malignant tumor cells, including MDA-MB-231 cells, show ERK activation when they are cultured at a high density [40,41]. Our data demonstrate that although ERK was not inhibited in L-132 cells, ERK was not activated in L-132 cells in a cell density-dependent manner; however, ERK was activated in A549 cells according to the cell density increment. We suggest this is the reason why inhibition of ERK activation induced by BDS-II only affected A549 cell migration and invasion even though ERK activation was inhibited in both A549 and L-132 cells cultured at a high density. In addition, although Kv3.1 and Kv3.4 were not cell density-dependently increased in L-132 cells, BDS-II inhibited ERK activation in L-132 cells cultured at a high density. Therefore, we concluded that BDS-sensitive Kv channels, including Kv3.1 and Kv3.4, are important and are related to ERK activation when the cells reach a high density, and only cancer cells, including A549 cells, at a high density utilize this relationship to coordinate migration and invasion. We think this might be the reason that Kv3.1, Kv3.4 and ERK activation are increased in a cell density-dependent manner only in A549 cells. Because cell-cell contact inhibition is an important regulatory mechanism of cell growth, the loss of cell-cell contact inhibition through ERK activation may be related to an invasive tumor phenotype [40], and the BDS effect that we observed may be obtained by inhibition of this tumorigenic property.

In summary, tumor hypoxia-related oxygen-sensitive Kv channels, including Kv3.1 and Kv3.4, were increased in a cell density-dependent manner in three representative tumor cell lines but not in L-132 cells, a normal cell line. BDS, which blocks the channels of the Kv3 subfamily, efficiently inhibited tumor cell migration and invasion but had no effect on tumor cell proliferation or normal cell migration and invasion. In addition, ERK activation and alterations in the diameter of acidic compartments, which were observed in a cell density-dependent manner in A549 cells, were inhibited by BDS, and inhibition of these cell density-dependent changes may be the key mechanism by which BDS inhibits tumor cell migration and invasion. These data indicate that Kv3.1 and Kv3.4 may be new therapeutic targets for cancer metastasis.

## 4. Materials and Methods

### 4.1. Cell Culture

Cells were cultured in RPMI 1640 (A549, MDA-MB-231, and HT-29 cells (Welgene, Daegu, Korea)) or DMEM (L-132 cells) containing NaHCO_3_ supplemented with 10% fetal bovine serum (FBS) and 1% antibiotic-antimycotic solution (Sigma, St. Louis, MO, USA) at 37 °C with 5% CO_2_. When the cells exhibited sufficient growth in a T75 flask (SPL Life Sciences, Pocheon-si, Gyeonggi-do, Korea), they were divided into various culture dishes or culture plates (SPL). Each cell line was then grown until cells reached the desired confluence for experiments (low, medium, or high). BDS-II treatments were performed after 18 to 24 h of incubation time for cell adherence. For the experiments, we seeded cells, and after the cells grew to an appropriate cell density, we took a photo and evaluated the percentage of the surface area of the plate or dish covered by the cells using ImageJ software (National Institutes of Health, Bethesda, MD, USA). Low density represents 20~30% cell confluence, medium density represents 40~60% cell confluence, and high density represents over 80% cell confluence. Cell seeding numbers are shown in Table 1, and 6-well plates were used for all experiments except the PCR analysis (100-mm dish) and the migration and invasion assays (manufacturer’s kit inserts were used).

### 4.2. Reverse Transcription-Polymerase Chain Reaction (RT-PCR)

RNA preparation was performed using Hybrid-R^TM^ (GeneAll, Seoul, Korea) according to the manufacturer’s instructions. Isolated RNA (1 μg) with random hexamers and M-MLV (Promega, Madison, WI, USA) was used to synthesize cDNA. The PCR reaction was performed with 2 μL of cDNA, 1× GoTaq^®^ green master mix (Promega), and specific target primers (Table 2) under the following reaction conditions: initial denaturation at 94 °C for 5 min and then 35 cycles of 94 °C for 40 s, each of the annealing temperatures for 40 s, 72 °C for 1 min, and an extension at 72 °C for 1 min, followed by a final extension at 72 °C for 7 min. The PCR products were loaded on 1.6% agarose gel for electrophoresis and analyzed with an ABI Prism 3730 XL DNA Analyzer (Applied Biosystems, Foster City, CA, USA) to confirm the channel mRNA expression in each of the three cancer cells.

### 4.3. Western Blotting

Cells were lysed using radio-immunoprecipitation assay (RIPA) buffer (Sigma), and the total protein concentration was measured with a bicinchoninic acid (BCA) protein assay kit (Pierce, Rockford, IL, USA). The quantified protein was loaded on a 10% acrylamide gel for sodium dodecyl sulfate polyacrylamide gel electrophoresis (SDS-PAGE) and then transferred to a nitrocellulose membrane (Whatman, Maidstone, Kent, UK). Then, 1× TBS-Tween 20 containing 5% nonfat milk (Difco, Franklin Lakes, NJ, USA) was used to block non-specific antibody binding, and protein-transferred membranes were probed overnight with commercially purchased primary antibodies targeting the proteins HIF-1α, Kv3.1, Kv3.3 (Abcam, Cambridge, MA, USA), Kv3.4 (Alomone labs, Jerusalem, Israel), tERK, pERK (Cell Signaling Technology, Inc., Danvers, MA, USA), β-actin, or vinculin (Santa Cruz Biotechnology, Finnell St., Dallas, TX, USA). Membranes probed with primary antibodies were incubated with horseradish peroxidase–conjugated goat, anti-rabbit or anti-mouse secondary antibody (GenDEPOT, Barker, TX, USA) for 1 h and visualized using a WesternBright™ Quantum™ (Advansta, Menlo Park, CA, USA). An ImageQuant LAS 4000 image analyzer (GE Healthcare Life Sciences, Songdo, Korea) was used to visualize immuno-complexes, and ImageJ software (National Institutes of Health) was used to quantify the data.

### 4.4. ROS Detection

ROS levels were analyzed using 2′,7′-dichlorodihydrofluorescein diacetate (H2DCFDA) (Invitrogen, Carlsbad, CA, USA). Cells were plated at different cell densities, cultured until they reached an appropriate cell density, and then washed twice with DPBS followed by a 30 min incubation with 37 °C DPBS containing 5 μM H2DCFDA. Finally, the cells were incubated for 15 min with fresh medium to allow recovery. Fluorescence images were taken using an EVOStm fl Digital Inverted Fluorescence Microscope (Fisher Scientific, Paisley, UK). The images were analyzed using ImageJ software (National Institutes of Health, Bethesda, MD, USA).

### 4.5. Cell Proliferation Assay

Cell proliferation was measured using an MTT assay (Sigma). Cells seeded in a 96-well plate were incubated with 5 mg/mL MTT solution for 2 h. After the incubation, the formazan crystals in each well were dissolved in 100 μL of dimethyl sulfoxide (DMSO), and the absorbance was measured at 570 nm. In addition to the MTT assay, the proliferation of cells seeded in a 6-well plate was visualized with Hemacolor^®^ rapid staining (Millipore, Billerica, MA, USA) according to the manufacturer’s instructions. Each of the three Hemacolor^®^ rapid staining solutions was applied for 1 min to stain the cells. Cells were washed with DPBS after the Hemacolor^®^ rapid staining.

### 4.6. Cell Migration and Invasion Assay

Cell migration was tested with a 2-well culture insert in a 35-mm dish purchased from ibidi (ibidi, Martinsried, Germany) according to the manufacturer’s instructions. Briefly, an appropriate number of A549 (5 × 10^4^), MDA-MB-231 (7 × 10^4^), HT-29 (2 × 10^5^), or L-132 (5 × 10^4^) cells was seeded in the 2-well culture insert and incubated for one day. After the culture-insert was removed, the cells were incubated again to observe their migration to the empty space in the well.

Cell invasion was confirmed with a 24-well culture insert (SPL) for a transwell invasion assay, which is also known as a Boyden chamber assay. A549 (7 × 10^4^), MDA-MB-231 (7 × 10^4^) or L-132 (7 × 10^4^) cells were placed on the upper layer of a cell-permeable membrane with FBS-free medium, and medium containing 10% FBS was placed below the cell-permeable membrane. Following a 24 h incubation, the cells that migrated through the membrane were stained via Hemacolor^®^ rapid staining (Millipore) and counted using ImageJ software (National Institutes of Health).

### 4.7. Acridine Orange Staining

A low or high density of A549 and L-132 cells was seeded in a 35-mm confocal dish (SPL), and the seeding cell density was same used in 6-well plates (Table 1). After 24 h of incubation, BDS-II treatment was applied until the cells reached the required cell density (approximately 30 h). Cells were incubated with acridine orange (2 μg/mL) (Sigma, St. Louis, MO, USA) for 15 min and then washed twice using prewarmed DPBS, and the plate containing the cells was filled again with 2 mL of DPBS. Fluorescence images were acquired with an LSM710 confocal microscope (Carl Zeiss, Hallbergmoos, Germany). The acridine orange signal was detected after 488 nm excitation at an emission wavelength of 543 nm for monomer type or 633 nm for dimer type. The images were analyzed using ImageJ software (National Institutes of Health, Bethesda, MD, USA).

### 4.8. siRNA Transfection

Cells were transfected with 60 nM siRNA-Kv3.1 or siRNA-Kv3.4 (Santa Cruz Biotechnology) and Lipofectamine™ 3000 reagent (Invitrogen) following the manufacturer’s instructions for adherent cells. Mock control and control siRNA transfection (Santa Cruz Biotechnology) were used as negative controls. Briefly, the A549 cells (1 × 10^5^) were plated in 6-well plates and incubated for 24 h prior to the transfection step in RPMI 1640 (Welgene, Daegu, Korea) containing 10% FBS without antibiotics. After 24 h of incubation, the cells were transfected using siRNA-Kv3.1 or siRNA-Kv3.4. After 48 h of transfection, the transfected cells were transferred to the cell migration kit and cell invasion kit to confirm the cell migration and invasion. The transfected cells were also transferred to a new 6-well plate at high density to confirm ERK activation.

### 4.9. Statistical Analysis

All data are shown as the mean ± standard error (SE). Student’s *t*-test was applied for statistical analysis of two groups of data, and one-way ANOVA with Tukey’s post hoc test was used for more than two groups of data. Two-way ANOVA was used for analysis of the acridine orange staining data in Figure 6 (GraphPad Prism version 5.0, GraphPad Software, La Jalla, USA).

## Figures and Tables

**Table 1:** Cell seeding numbers.

Culture Place	Cell Name	Cell Density	Number of Cells	Culture Time
6-well plate	A549	low	2 × 10^4^	2~3 days
		med	4 × 10^4^	
		high	1.2 × 10^5^	
	MDA-MB-231	low	2 × 10^4^	
		med	4 × 10^4^	
		high	1.2 × 10^5^	
	HT-29	low	5 × 10^4^	
		med	1.5 × 10^5^	
		high	5 × 10^5^	
	L-132	low	1 × 10^4^	
		med	4 × 10^4^	
		high	1.2 × 10^5^	
100 mm dish	A549	low	2 × 10^5^	2~3 days
		med	4 × 10^5^	
		high	1 × 10^6^	
	MDA-MB-231	low	2 × 10^5^	
		med	4 × 10^5^	
		high	1 × 10^6^	
	HT-29	low	5 × 10^5^	
		med	1.5 × 10^6^	
		high	1 × 10^7^	

**Table 2:** RT-PCR primers.

Subtype	Accession No.	Size (bp)	Primer Sequence (Forward/Reverse)	Annealing (°C)
Kv1.2	L02752	513	5′-GGGACAGAGTTGGCTGAGAA-3′	60
			5′-GGAGGATGGGATCTTTGGAC-3′	
Kv1.5	M55513	917	5′-TGCGTCATCTGGTTCACCTTCG-3′	60
			5′-TGTTCAGCAAGCCTCCCATTCC-3′	
Kv2.1	L02840	451	5′-GGAAGCCTGCTGTCTTCTTG-3′	65
			5′-CTTCATCTGAGAGCCCAAGG-3′	
Kv3.1	S56770	550	5′-AACCCCATCGTGAACAAGACGG-3′	60
			5′-TCATGGTGACCACGGCCCA-3′	
Kv3.3	AF055989	284	5′-CCTCATCTCCATCACCACCT-3′	60
			5′-CGAGATAGAAGGGCAGGATG-3′	
Kv3.4	M64676	631	5′-TTCAAGCTCACACGCCACTTCG-3′	65
			5′-TGCCAAATCCCAAGGTCTGAGG-3′	
Kv4.2	NM_012281.2	157	5′-GCCTTCTTCTGCTTGGACAC-3′	60
			5′-TCATCACCAGCCCAATGTAA-3′	
Kv9.3	AF043472	395	5′-CTGGGGAAGCTGCTTACTTG-3′	60
			5′-CAGATTTTCTTCCGGAGCTG-3′	

## Supplementary Materials

Supplementary materials can be found at http://www.mdpi.com/1422-0067/19/4/1061/s1.

## Abbreviations

Kv channels	voltage-gated potassium channels
ROS	reactive oxygen species
BDS	blood depressing substance
ERK	extracellular signal-regulated kinase
