# Supplementary figures and images for: Kv3.1 and Kv3.4, Are Involved in Cancer Cell Migration and Invasion
Source: Int J Mol Sci. 2018 Apr 2;19(4):1061. doi: 10.3390/ijms19041061 (PMC5979479; doi:10.3390/ijms19041061)

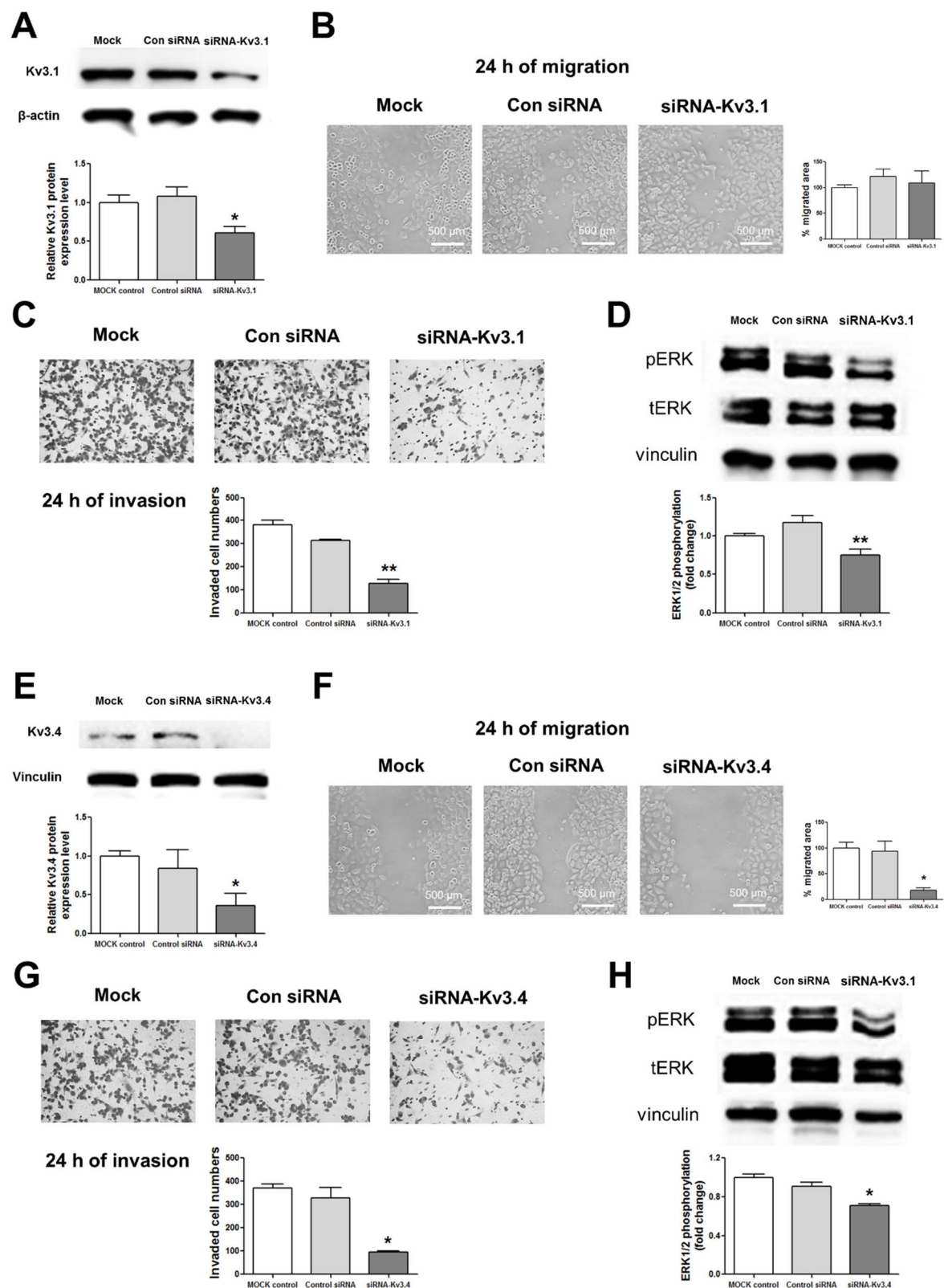

Supplement: Supplementary file 1 [file ijms-19-01061-s001.pdf]
